# An azoospermic male with a novel chromosome 46, XX, der(15)t(Y; 15)(p11.3; p12)

**DOI:** 10.1002/ccr3.5984

**Published:** 2022-07-11

**Authors:** Jiebin Wu, Guanli Hu, Jingfang Zhai, Conghui Han, Zhenbei Li

**Affiliations:** ^1^ Department of Prenatal Diagnosis Medical Center Xuzhou Central Hospital, Xuzhou Clinical Schools of Nanjing Medical University and Xuzhou Medical University Xuzhou Jiangsu China; ^2^ Department of Urology Xuzhou Central Hospital, Xuzhou Clinical Schools of Nanjing Medical University and Xuzhou Medical University Xuzhou Jiangsu China

**Keywords:** 46, XX male, azoospermia, cytogenetics, molecular genetics, positive SRY gene

## Abstract

Male individuals with a 46, XX karyotype are commonly diagnosed with 46, XX male sex reversal syndrome, one of the rarest sex chromosomal anomalies. In this case, we report a rare XX male with Y‐specific DNA sequences located near the end of chromosome 15 p‐arm, which was verified by fluorescent in situ hybridization (FISH) as well as copy number variation sequencing (CNV‐seq) based on the next‐ generation sequencing method (>100 Kb). To the best of our knowledge, there have been no reports of XX male with the Yp region transferred to the terminal of chromosome 15 short arm.

## Abstract 2

We report an azoospermia male with a novel chromosome 46, xx, der(15)t(Y; 15)(p11.3; p12) by CNV‐seq combined with traditional karyotype analysis and FISH. To the best of our knowledge, there have been no reports of XX male with the Yp region transferred to the terminal of chromosome 15 short arm.

## 1 INTRODUCTION

46, XX male sex reversal syndrome is characterized by gonadal dysplasia after puberty, azoospermia, sterility, high‐level gonadotropin, and sex chromosome aberration, which was first defined and reported in 1964.^1^ Some studies have illustrated that about 80%–90% of Xp‐Yp translocations can account for 46, XX SRY positive male sexual inversion syndrome.^2^, ^3^ The atypical localizations of the sex‐determining region Y (SRY) gene have been reported at the terminal of the long arm of chromosome X, 1, and 16,^4^, ^5^, ^6^ also occasionally at the short arm of chromosomes 3, 6, 14, and 18.^7^, ^8^, ^9^, ^10^

The incidence of XX males in humans is 1 in 20,000–30,000 male newborns.^11^ XX males can be divided into SRY positive group and SRY negative group according to the presence of the SRY gene.^12^ Approximately 90% reported XX males possess the SRY positive gene, which is critical for the encoding of the testis‐determining factor (TDF).^13^, ^14^ During the first meiosis period of paternal germ cells, the sperm containing SRY gene is produced from the unbalanced translocation between the Y chromosome and other chromosomes. The sperm then combines with a normal oocyte to produce an abnormal zygote, resulting in a male phenotype and a female karyotype. These XX individuals with SRY‐positive gene typically exhibit a masculine phenotype at birth and have normal external genitalia, although rare exceptions have been reported.^15^ However, the translocation between Y chromosome and acrocentric chromosome is rare, the previous studies revealed that different individuals with SRY‐positive gene presented diverse manifestations: oligospermia in a 32‐year‐old man with a Yp/13p translocation,^16^ and azoospermia in a 23‐year‐old man with the karyotype 46,XX, t(Y;15)(q12;p11), whereas his father in the latter case was fertile carrying de novo derivative chromosome 15 [45,X, t(Y;15)(q12;p11)].^17^ Herein, we report a male with normal phenotype, infertility, and azoospermia carrying a chromosomal constitution of 46,XX,der(15)t(Y;15)(p11.3;p12). The presence of the SRY gene was confirmed with FISH. All analyzed sequence‐tagged site (STS) markers in the azoospermia factor (AZF) region were absent at the Y chromosome.

## 2 MATERIALS AND METHODS

### 2.1 Case presentation

A 32‐year‐old young man (social gender) and his wife were referred to the urological surgical department of Xuzhou Central Hospital for infertility, after 2 years of marriage with reproductive behavior. He was a phenotypically normal male, with a height of 175 cm and a weight of 75 kg. The clinical examination showed that his prostate, male hair distribution, and breast were normal, while bilateral testes were smaller than normal, and azoospermia was confirmed through three semen analysis according to the World Health Organization guidelines.^18^ Endocrine analysis showed elevated serum hormone levels: luteinizing hormone (LH) 21.92 mIU/ml (normal range [NR]: 1.24–8.62) and follicle‐stimulating hormone (FSH) 34.82 mIU/ml (NR: 1.27–19.26), whereas estradiol, testosterone, and prolactin levels were normal. Ultrasound showed the bilateral small testes were both 14 × 10 × 10 mm in size, while the sizes and echoes of bilateral kidneys, ureters, bladder, and prostates were normal. A testosterone replacement therapy was recommended. His parents were fertile with a son and a younger daughter. His wife's ovulation was normal according to ultrasonic evaluation and endocrine tests. It is worth mentioning that his wife has a healthy daughter with her ex‐husband.

### 2.2 Cytogenetic analysis

Routine chromosome preparation from peripheral blood lymphocytes was carried out for the couple, his father, and his younger sister by G‐banding according to the standard protocols. The reports were analyzed in line with the International System for Human Cytogenetic Nomenclature at the level of 300–400 bands (ISCN2020).

### 2.3 FISH

FISH on the 20 metaphase chromosomes of cultured peripheral blood lymphocytes of the young man was combined with DAPI banding analysis, using centromeric probe DXZ1 (Xp11.1‐q11.1), an SRY‐specific probe (SRY, Yp11.3) following the instructions provided by the manufacturer (Vysis).

### 2.4 CNV‐seq

Uncultured genomic DNA was extracted from the sample. The following entire operation process of CNV‐seq included DNA fragmentation, DNA libraries' construction, massive sequencing in parallel, and the raw reads' being conducted. Finally, the results of data were assessed according to the American College of Medical Genetics (ACMG) standards and guidelines.^19^

### 2.5 Detection of AZF microdeletions

Genomic DNA extracted from 200 μl peripheral blood lymphocytes of the young man was used to perform real‐time PCR to detect AZF microdeletions and the SRY genes. Two STSs were chosen for each AZF subregion (AZFa SY84, SY86; AZFb SY127, SY134, and AZFc SY254, SY255 loci) and the SRY gene following the manufacturer's instructions (Toujing Life Technology Co., Ltd). The DNA samples of normal man, normal woman, and double distilled water were used as positive, negative, and blank controls, respectively.

## 3 RESULTS

**FIGURE 1:**
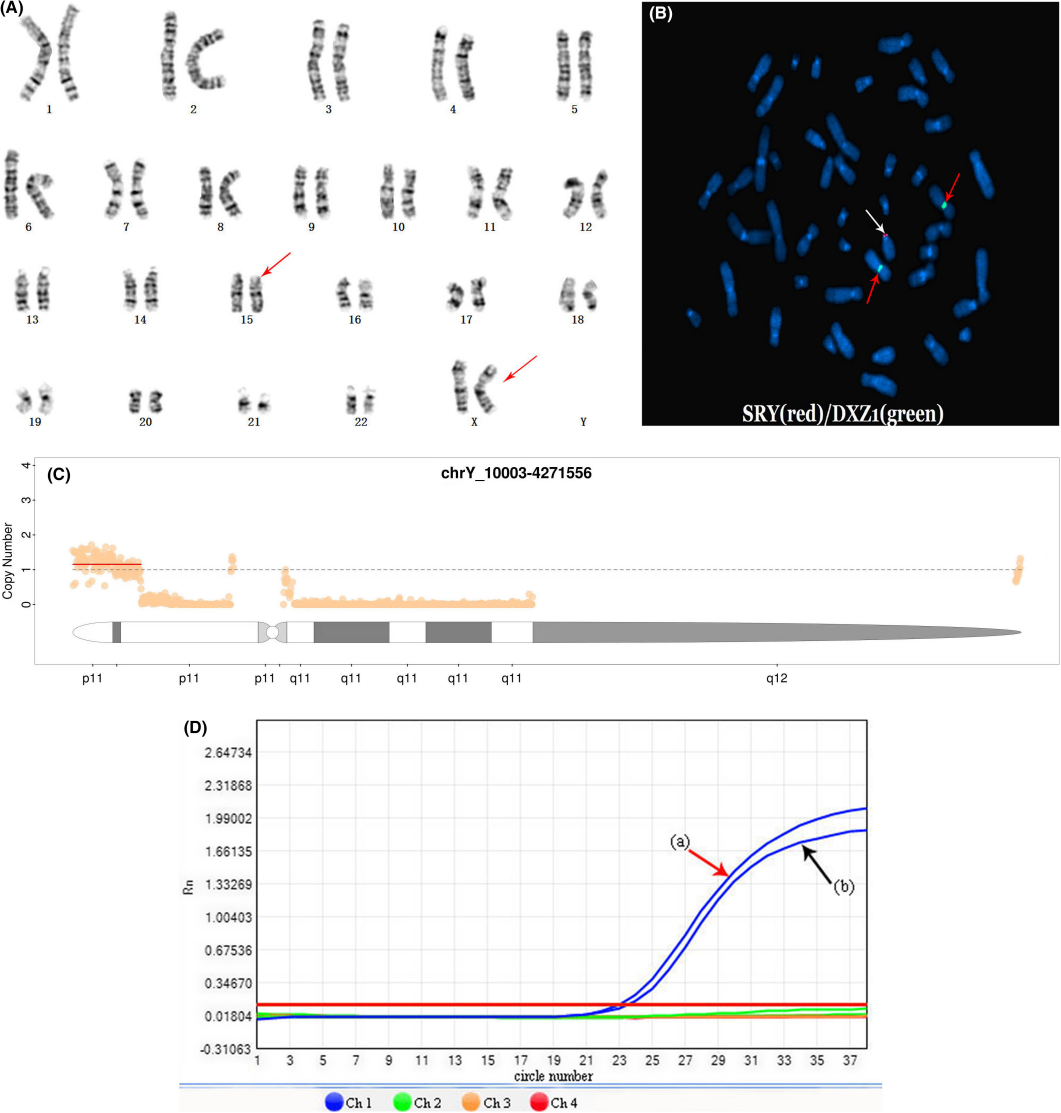
(A) G‐banded karyotype of a peripheral blood sample of the young man: 46, XX. (B) FISH results: 46, XX, ish der(15)t(Y;15)(p11.3;p12)(SRY+). Red arrows indicated two X chromosome centromeric signals, and white arrow showed one SRY specific signal on the distal tip of the short arm of chromosome 15. (C) CNV‐seq result: an approximately 4.262‐Mb fragments presented in Yp11.32‐p11.3 (chrY: 10003–4,271,566). (D) Green, yellow, and red amplification curves indicated that the AZFa (sY84, sY86), AZFb (sY127, sY134), and AZFc (sY254, sY255) were absented, and the SRY gene (A) and ZFX/ZFY (B) was existing

In this study, the young man had the female karyotype: 46, XX (Figure 1A). The karyotypes of his wife, his father, and his younger sister were normal. The FISH result showed the 46,XX,ish der(15)t(Y;15)(p11.3;p12) (Figure 1B) and confirmed the presence of the SRY gene on the distal tip of the short arm of chromosome 15. The CNV‐seq result illustrated that only an approximately 4.262‐Mb fragment was presented in Yp11.32‐p11.3 (chrY: 10003–4,271,566) (Figure 1C). The AZF microdeletion detection displayed the absences of AZFa, AZFb, and AZFc on Y chromosome and the existence of SRY gene (Figure 1D).

## 4 DISCUSSION

46,XX male sex reversal syndrome, characterized by discordant gonads and sex chromosomes, is a rare sex chromosome abnormality which can lead to infertility. Most of the patients are diagnosed through genetic evaluation and related testing after puberty due to abnormal male phenotypes and infertility. The clinical manifestations of 46, XX male sex reversal syndrome vary with the presence or absence of SRY gene, involving some common features such as small testes, azoospermia, oligospermia, the differences in male phenotype, external genitalia, and female breast development.^14^, ^20^, ^21^ In this case, clinical examinations revealed that the adult was a phenotypically normal male with bilateral smaller testes and azoospermia. Furthermore, the endocrine analysis identified the high levels of gonadotropin hormones, and the karyotype analysis combined with FISH revealed the man's de novel karyotype: 46, XX,ish der(15)t(Y;15)(p11.3;p12)(SRY+). CNV‐seq helped us clarify an approximately 4.262‐Mb fragment in Yp11.32‐p11.3. Thus, we made it clear that the complex rearrangement illustrated the absence of Yq region and a copy of the Yp11.32‐p11.3 (chrY: 10003–4,271,566) region was translocated to the short arm of chromosome 15 (Figure 1C). In addition, Figure 1C shows two other small copy‐number peaks: the centromere and its adjacent fragment located in the heterochromatin region contain highly repetitive sequences, resulting in poor sequencing quality and local changes of fluctuation value which make it difficult to determine the specific copy number. Hence, the karyotype analysis combined with FISH and CNV‐seq can help us to further clarify the structure of chromosomes. It should be emphasized that about 828‐bp SRY gene (OMIM 480000) located in Yp11.3 and currently is considered to be the best candidate gene for TDF.^4^ The presence of SRY causes the bipotential gonad to develop into testis, which plays a key role in determining human sex. The presence of the SRY gene determines the young man's normal male appearance with bilateral smaller testes caused by the lower level of testosterone hormones, which lead to the negative feedback effect—the higher levels of LH and FSH.^2^, ^14^, ^22^ The patients with gonadal dysplasia are deficient in permatogenesis; however, they still have certain compensatory endocrine alterations to maintain the male phenotype and sexual behavior in adulthood. In addition, there is an absence of the Yq in our patient. The Yq fragment anchors the AZF genes and plays a critical role in the process of spermatogenesis. In our patient, the mechanism is relatively clear that Yq absence causes azoospermia and infertility.^23^, ^24^ AZF is located on chromosome Yq11. The Yq fragment leading to spermatogenesis is divided into AZFa, AZFb, and AZFc regions. The absences of AZFa, AZFb, and AZFc are prone to lead to different degrees of spermatogenic disorder and infertility.

As was shown, the AZF microdeletion and SRY gene detection by real‐time PCR indicated that the AZFa (sY84, sY86), AZFb (sY127, sY134), and AZFc (sY254, sY255) were absent and the presence of positive SRY was verified. Here, real‐time PCR is recommended for the detection of AZF microdeletions and the SRY genes due to its rapid, high efficiency, automatic, cost‐effective, and repeatable, and is useful for clinicians to evaluate the patient's relevant genetical condition of chromosome Y.^25^

In terms of clinical treatment, if the patient does not need to change sex, treatment should focus on the genitourinary malformations' correction and timely androgen replacement therapy to promote the healthy development of male secondary sexual characteristics.^2^, ^22^ Based on the evaluations of general genetic and endocrine results, we selected the timely androgen replacement therapy for the adult. We also recommended that the couple should either choose assisted reproductive technology of sperm donation to carry in vitro fertilization and embryo transfer (IVF‐ET) or adopt a child to help them obtain offspring.

In conclusion, 46, XX, sex reversal syndrome, as a relatively rare sexual dysplasia, has brought more challenges to clinical diagnosis and treatment due to its high heterogeneity of clinical manifestations and diversity of pathogenesis. In addition to the clinical history, the whole genome copy number variation detection combined with traditional karyotype analysis, as well as FISH may be an effective approach to confirm the genetic condition. Meanwhile, real‐time PCR is recommended for clinicians to detect AZF microdeletions and the SRY gene to help clinicians evaluate the patient's spermatogenesis related to chromosome Y. In a word, the comprehensive assessments may be conducive to the diagnosis, classification, and clinical rational treatment for 46, XX, sex reversal syndrome.

## AUTHOR CONTRIBUTIONS

Jingfang Zhai and Conghui Han conceived the project. Jiebin Wu, Guanli Hu, and Jingfang Zhai participated in the recruitment, clinical information acquisition, and comprehensive evaluation of the male and their families, collaborated in the molecular analyses, and wrote the manuscript. Zhenbei Li performed cytogenetics analysis. All authors were involved in revising the manuscript.

## CONFLICT OF INTEREST

The authors declare no conflict of interest.

## ETHICAL APPROVAL

The present study was approved by the Xuzhou Central Hospital ethics committee (No. XZXY‐LJ‐20190210‐037). The evaluation of the young man was performed after receiving written informed consent.

## CONSENT

Written informed consent was obtained from the patient in accordance with the journal's patient consent policy.

## Data Availability

The data of this study are available in the department of Prenatal Diagnosis Medical Center, Xuzhou Central Hospital.
